# Gene regulatory networks analysis of muscle-invasive bladder cancer subtypes using differential graphical model

**DOI:** 10.1186/s12864-021-08113-z

**Published:** 2021-12-01

**Authors:** Yongqing Zhang, Qingyuan Chen, Meiqin Gong, Yuanqi Zeng, Dongrui Gao

**Affiliations:** 1 School of Computer Science, Chengdu University of Information Technology, Chengdu, 610225 China; 2 School of Computer Science and Engineering, University of Electronic Science and Technology of China, Chengdu, 611731 China; 3 West China Second University Hospital, Sichuan University, Chengdu, 610041 China; 4 School of life Science and technology, center for information in medicine, University of Electronic Science and Technology of China, Chengdu, 611731 China

**Keywords:** Muscle-invasive bladder cancer, Molecular subtypes, Gene regulatory networks, Differential networks

## Abstract

**Background:**

Recently, erdafitinib (Balversa), the first targeted therapy drug for genetic alteration, was approved to metastatic urothelial carcinoma. Cancer genomics research has been greatly encouraged. Currently, a large number of gene regulatory networks between different states have been constructed, which can reveal the difference states of genes. However, they have not been applied to the subtypes of Muscle-invasive bladder cancer (MIBC).

**Results:**

In this paper, we propose a method that construct gene regulatory networks under different molecular subtypes of MIBC, and analyse the regulatory differences between different molecular subtypes. Through differential expression analysis and the differential network analysis of the top 100 differential genes in the network, we find that SERPINI1, NOTUM, FGFR1 and other genes have significant differences in expression and regulatory relationship between MIBC subtypes.

**Conclusions:**

Furthermore, pathway enrichment analysis and differential network analysis demonstrate that Neuroactive ligand-receptor interaction and Cytokine-cytokine receptor interaction are significantly enriched pathways, and the genes contained in them are significant diversity in the subtypes of bladder cancer.

## Background

In recent years, five immunotherapy drugs have been approved to bladder cancer, and erdafitinib which is the first targeted drug for gene alternation was approved. Targeted therapy of cancer is greatly encouraged, which contributes to achieve precision medicine. Muscle-invasive bladder cancer (MIBC) is a highly heterogeneous malignant tumor, whose prognosis and survival rates among molecular subtypes are significantly different. Currently, Gene regulatory networks(GRNs) under different pathological conditions have been constructed to reveal the regulatory differences, but they have not been applied to different subtypes of bladder cancer. The motivation of our study is to construct differential networks among subtypes of MIBC, which reveals the gene regulatory differences between the subtypes.

Gene regulatory network is a kind of biological network that expresses complex regulatory relationships between genes,and is meaningful in medical diagnosis, treatment, and drug design [1]. Gaussian Graphical Models (GGMs) are widely used in estimating GRNs [2]. It assumes that the gene expression measurements follow a multivariate Gaussian distribution, so the precision matrix or inverse covariance matrix can reflect the conditional dependence between genes. This method belongs to the “reverse engineering” problem of gene regulatory network. A gene regulatory network model with the known real expression data is established to be well consistent with the real data, so as to the potential regulatory relationship can be inferred. Reverse engineering is one of the important methods for constructing gene regulatory networks.

Gene regulatory networks are dynamic in time and space [3–5]. In particular, more researchers focus on the difference between conditions in GRNs, and pay attention to the variety of regulatory relationships and find key genes with significant change. Therefore, methods of constructing differential network directly from the expression data of two conditions are proposed. Zhang and Zou [6] proposed D-trace method to estimate differential network directly, which is more natural and convenient than classical log-likelihood function. Tian [7] and Yuan [8] add L1 penalty on the D-trace loss function to estimate the precision matrix of sparse differential networks.With the development of high-throughput sequencing technology and computational biology, plenty of static gene regulation data have been collected and summarized, such as Transcriptional Regulatory Relationships Unraveled by Sentence-based Text-mining (TRRUST) database [9], which provides useful information to construct GRNs. Furthermore, the authors in [10] integrate static regulatory data and gene expression data to reconstruct GRNs, called WD-trace. Expect the observed variable from samples, gene regulation may also be affected by other latent or unobserved factors, such as miRNA. Taking the latent variables into account, Ouyang [11] proposed a new model JDNA jointly estimates multiple differential networks with latent variables from multiple data sets. The model is also based on the D-trace loss function, the difference is that two new penalty functions, group lasso penalty and fused lasso penalty, are applied to learn the common support of difference edges on multiple data sets or make full use of the similarities. To reveal how gene regulatory networks change over cancer development, Xu [12] proposed the tDNA model to jointly estimate multiple time-varying differential networks. They designed a tree-structured group lasso penalty to identify the common and specific hub nodes on differential networks.

**Fig. 1:**
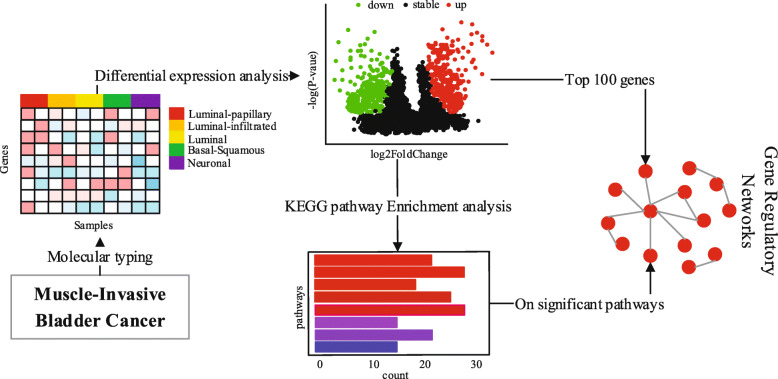
The whole analysis flow of Muscle-invasive bladder cancer gene regulatory networks

Inspired by the differential network and molecular subtypes of bladder cancer, we apply the WD-trace method to construct differential networks in different subtypes of bladder cancer. Then, we analyze the regulatory differences between two subtypes. According to the molecular typing results of [13], we download and preprocess the subtypes data, and preform differential expression analysis. After differential expression analysis, we firstly construct differential networks from first 100 gene with significant differences between two subtypes. In order to identify biological pathways where differential genes play a key role, we perform Kyoto Encyclopedia of Genes and Genomes (KEGG) [14] pathway enrichment analysis of differential genes.From the results of differential gene enrichment, we can see that the significant enrichment pathways between each couple of subtypes contain one or two pathways of Neuroactive ligand-receptor interaction and Cytokine-cytokine receptor interaction, thus we construct differential network based on these two pathways. The whole analysis flow of MIBC gene regulatory network is shown in Fig. 1.

Our contribution can be summarized as follows:

1)A complete process of muscle-invasive bladder cancer gene regulatory network analysis was proposed, including differential expression analysis, functional enrichment analysis and differential network construction. This analysis process observes the differences between molecular subtypes from multiple perspectives.

2)It was found that some genes not only have significant differences in gene expression between molecular subtypes, but also have significant differences in regulatory relationships. For example, SERPINI1, NOTUM and FGFR1 have some related to the cancer biomarker.

3)Gene regulation on two biological pathways have been found to be significantly different between molecular subtypes, where Cytokine-cytokine receptor interaction has been shown to be related to bladder cancer. Studying on these two pathways has important implications for the pathogenesis of different subtypes.

## Results

### Results of the differential networks of top 100 differential gene

Here, differential expression analysis was performed between any two subtypes of five subtypes, and the thresholds *p*_*adjust*<0.01 and *log*2*FoldChange*>2 were set to obtain differentially expressed genes. Ten comparisons with five subtypes were made, four comparison results are shown in Fig. 3 which the green and red points in the plot represent the differential genes, respectively. From Fig. 3, it can be observed that the number of differential genes in Luminal-papillary and Luminal subtypes, Luminal-infiltrated and Luminal subtypes is less than that of any other two subtypes, which indicates that these two subtypes are more similar to Luminal subtype. Furthermore, it also presents that the Luminal-infiltrated, Luminal-papillary, and Luminal subtypes are isolated from the early identified Luminal subtypes in [15].

To reveal the difference of gene regulation among subtypes, we constructed differential regulatory networks on the top 100 differential genes obtained from differential expression analysis. Figure 4 presents the differential networks between Luminal and Neuronal subtype, which contains a large number of differential regulatory edges. Such a dense network indicates that the top 100 differential genes between the two subtypes have large differences in expression levels, and great changes have also taken place in regulatory relationship.

**Fig. 2:**
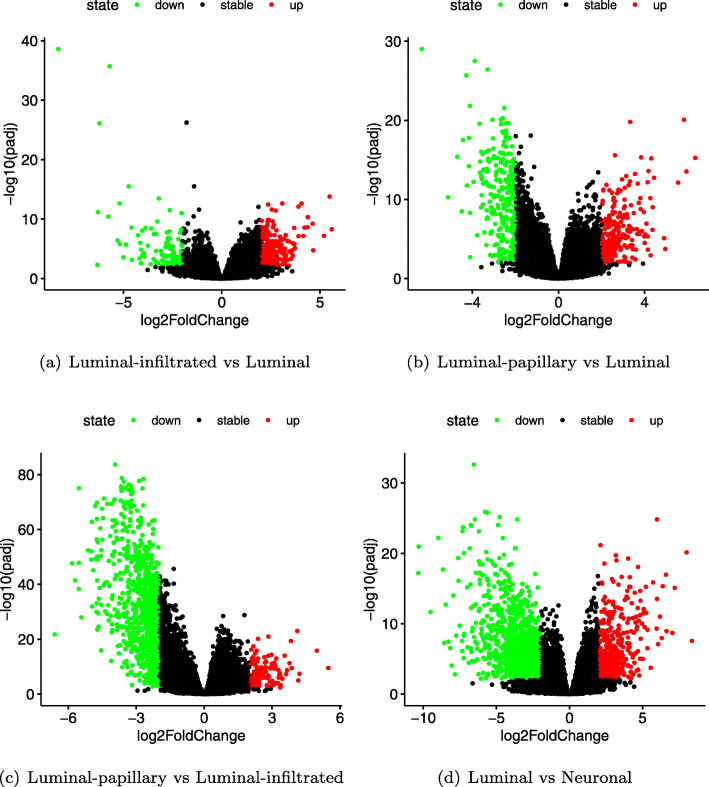
Several volcano plot of differential expression analysis among subtypes. The green and red points in a plot represent the differential genes between two subtypes screened by the threshold *p*_*adjust*<0.01 and *log*2*FoldChange*>2

**Fig. 3:**
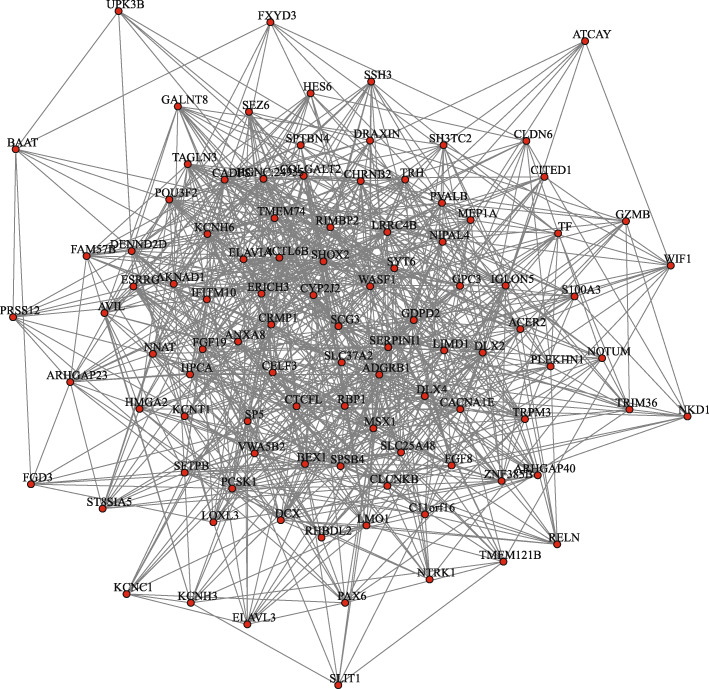
Differential networks with top 100 differential genes between Luminal and Neuronal subtype. The nodes belong to the top 100 differential genes obtained form differential expression analysis, and the edges are the regulatory differences between the two subtypes

**Fig. 4:**
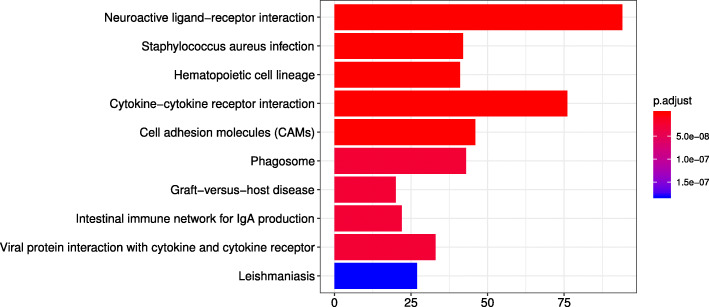
The top 10 significant enrichment pathways between Neuronal and Basal-Squamous subtype.The vertical axis indicates the pathways, the horizontal axis indicates the number of genes enriched in a pathway, and the color indicates the value of p.adjust

On the differential networks, some key genes were discovered. NOTUM may be a key gene of Neuronal subtype, because there are highly differentially expressed genes between NOTUM gene and other subtypes. NOTUM gene encodes Palmitoleoyl-Protein Carboxylesterase, which acts as a key negative regulator of the Wnt signaling pathway [16]. However, abnormal activation of the Wnt signaling pathway can lead to cell growth and development defects, and may lead to tumorigenesis. Numerous studies have shown that the abnormal Wnt signaling pathway is associated with many malignant tumors [17] [18], such as liver cancer, colorectal cancer, bladder cancer, cervical cancer. In the same time, there is a research showing that NOTUM is overexpressed in primary colorectal cancer, gastric cancer, liver cancer, breast cancer, lung cancer, ovarian cancer and endometrial cancer [19].

In addition, SERPINI1 is one of top 10 genes differentially expressed between Neuronal and all other subtypes. For Luminal and Neuronal subtypes, it ranks the 5th in the differential expression genes and the 1st in the differential network, which connected sides is 34. In other words, it can be concluded that the SERPINI1 gene regulation difference in these subtypes is significant. Besides, SERPINI1 gene has been reported to be involved in malignant tumors. For instance, it has been reported to be a genetic marker for the discovery of hepatocellular carcinoma [20] and may be one of the candidate biomarkers for the diagnosis of colorectal cancer [21].What’s more, some studies on gastric cancer have shown that SERPINI1 has a potential tumor suppressor function in the stomach [22]. In summary, SERPINI1 may be a marker to distinguish the Neuronal subtype.

Similarity, FGFR1 gene not only has significant differences in single gene expression, but also is a critical node in the differential network for the Luminal-papillary and Neuronal subtypes. Since erdatinib is a targeted drug for genetic alteration (FGFR). The regulatory relationship of FGFR in subtype level is helpful to predict the side effects of erdatinib. The statistical results of these key genes are summarized in Table 1. The log2FoldChange and p-adjust are the result of differential expression analysis, and the degree is the result of differential network. The higher the absolute value of log2FoldChange and the lower the p-adjust, the greater the expression difference between the two subtypes. Moreover, the degree of a gene in differential network indicates the number of differences in the regulatory relationship with other genes.

**Table 1:** The measurement of important genes in differential expression analysis and differential network

Genes	Subtypes	log2FoldChange	p-adjust	Degree
NOTUM	PN	-7.50	2.01E-69	3
	IN	-6.15	6.28E-42	7
	LN	-6.54	2.35E-33	13
	NB	7.00	2.22E-60	1
SERPINI1	PN	-3.79	7.82E-45	3
	IN	-3.70	1.61E-38	5
	LN	-3.57	1.50E-25	34
	NB	3.76	1.45E-44	1
FGFR1	PN	-4.26	2.48E-43	8

Note: P, I, L, B and N denote Luminal-papillary, Luminal-infiltrated, Luminal, Basal-Squamous, and Neuronal subtype separately, and PI denotes the data between Luminal-papillary and Luminal-infiltrated. log2FoldChange and p-adjust are the result of differential expression analysis, and degree is the result of differential network

Besides, we also performed Gene Ontology (GO) analysis on the top 100 differentially expressed genes to understand the function of these genes and their products in the biological processes. The top 100 differential genes between Luminal-papillary and Luminal-infiltrated and between Luminal-papillary and Luminal are both mainly enriched in the extracellular matrix organization, and a biological process in the assembly, arrangement of constituent parts, or disassembly of an extracellular matrix is discussed. The process affects cell behaviours, such as proliferation and migration, cell differentiation and death. Abnormal extracellular matrix dynamics can lead to deregulated cell proliferation and invasion, cell death, and cell differentiation. Furthermore, it can bring about pathological processes such as tissue fibrosis and cancer [23]. The differential genes enrichment results between the Neuronal subtype and other subtypes have the characteristics of the Neuronal subtype. In addition, the biological processes or molecular functions are mainly related to the development of neurons and signal transmission, such as neuron projection development and axon development.

### Results of the differential networks on pathways

**Fig. 5:**
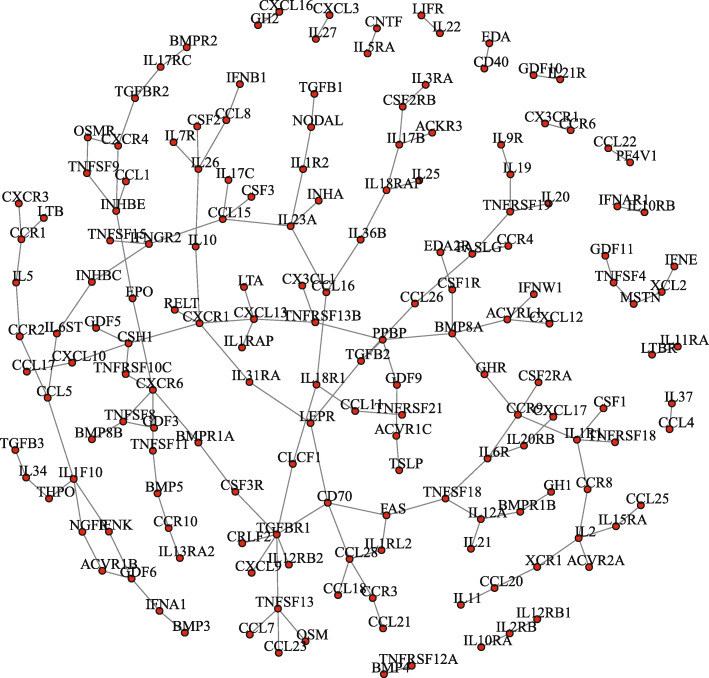
Differential networks on Cytokine-cytokine receptor interaction between Luminal and Neuronal. The node represents the gene on the pathway, and the edge is the regulatory differences between the two subtypes

A biological pathway is a series of actions among molecules in a cell which leads to a certain product or a change in the cell. Compared with the differential networks on the first 100 differential genes, the differential networks on a pathway can accurately locate differences and understand the changes of different subtypes in specific action. In order to obtain pathways related to the subtype differences, we performed KEGG enrichment analysis on differentially expressed genes. Figure 5 presents the results of top 10 significant enrichment pathways between Neuronal and Basal-Squamous subtype. The abnormality of these pathways may be related to the formation of Neuronal and Basal-Squamous subtype. By counting the significant enrichment pathways between all subtypes, we found that the significant enrichment pathways between each two subtypes in Neuroactive ligand-receptor interaction or Cytokine-cytokine receptor interaction, which are given in Table 2. In other words, these two pathways appear in the top 10 significant pathways ranked by p-adjust between all subtypes.

**Table 2:** KEGG pathway enrichment results of Neuroactive ligand-receptor interaction and Cytokine-cytokine receptor interaction

	Cytokine-cytokine receptor interaction			Neuroactive ligand-receptor interaction		
	*p*_value	p_adjust	count	*p*_value	p_adjust	count
PI	1.73E-10	1.49E-08	47	1.42E-05	3.31E-04	40
PL	-	-	-	3.12E-05	3.37E-03	22
PN	-	-	-	4.98E-19	1.45E-16	86
PB	4.33E-31	1.26E-28	103	2.76E-04	2.77E-03	56
IL	2.69E-04	2.20E-03	17	4.83E-04	3.67E-03	18
IN	3.96E-06	1.02E-04	46	6.73E-18	1.91E-15	76
IB	2.36E-04	6.14E-03	24	-	-	-
LN	4.14E-04	8.96E-03	39	2.74E-15	7.96E-13	70
LB	1.57E-26	4.61E-24	89	7.01E-05	6.65E-04	52
NB	1.44E-14	1.05E-12	76	1.25E-19	3.65E-17	93

Note: The count means that the number of differential genes between two subtypes annotated in the pathway

Cytokine-cytokine receptor interaction is an important pathway which contains a variety of cytokines and their receptors. The combination of cytokines and their receptors have an effect on cells, such as cell growth, proliferation and differentiation, and regulates the collective immune response. Besides, it not only provides data for the study of the pathogenesis of autoimmune diseases, tumors and immunodeficiency diseases at the cellular and molecular level, but also provides guidance for clinical treatment and diagnosis. Neuroactive ligand-receptor interaction consists of a group of neuroreceptor genes, such as dopamine receptor and proto oncogene, which are involved in environmental information processing and signal molecule interaction.

**Fig. 6:**
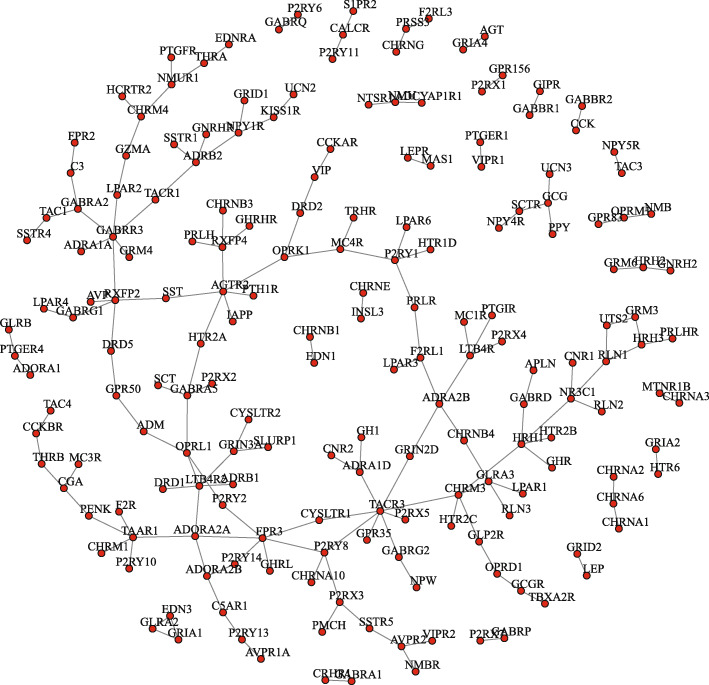
Differential networks on Neuroactive ligand-receptor interaction between Luminal-infiltrated and Basal-Squamous.The nodes represents the gene on the pathway, and the edge is the regulatory differences between the two subtypes

**Fig. 7:**
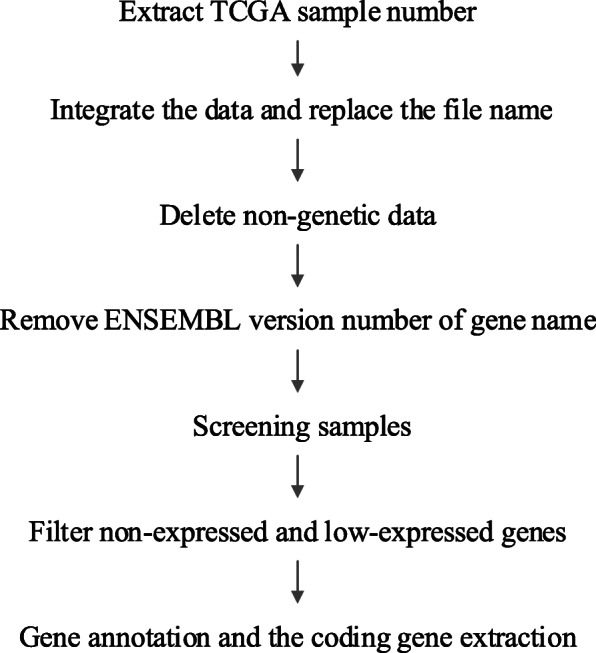
The workflow of data preprocessing in this work

In order to further determine whether a group of genes jointly affect the risk of disease characteristics in biological pathways, we construct differential networks of these two pathways. In the differential network, hub genes and other genes have regulatory relationship and can be directly displayed, and the regulatory differences among genes can also be captured. Figure 6 presents the differential network between Luminal and Neuronal based on Cytokine-cytokine receptor interaction. The pathway genes between the two subtypes changed in the regulatory relationship in Fig. 6. Additionally, Fig. 7 presents the differential network on Neuroactive ligand-receptor interaction between Luminal-infiltrated and Basal-Squamous.

For the differential networks on Cytokine-cytokine receptor interaction, the IL29RA gene between Luminal-papillary and Basal-Squamous is a hub gene, which encode protein interleukin 20 receptor subunit alpha protein and is a member of the type II interleukin receptor family. Recent studies have identified IL20RA is a novel risk gene for a variety of autoimmune diseases [24]. IL24 and its receptors regulate the growth and migration of pancreatic cancer cells, which is the potential biomarkers for IL24 molecular therapy [25]. In addition, IL24 receptor rarely intact expression in hepatoma cell lines [26]. The hub gene of Luminal-infiltrated and Basal-Squamous subtypes, IL36G, belongs to the IL-1 cytokine family. And IL36G has been identified to have anti-tumor effects in breast cancer and melanoma. What’s more, IL36G has been shown to enhance the effector function of immune cells such as NK cells, so that the environment is transformed into the tumor destruction. Finally, IL36G may contribute to the growth of anti-tumor and metastasis of tumor [27]. Furthermore, the hub gene FASLG of Luminal-papillary and Neuronal encodes the FasL protein, on the other hand, the prognostic value of soluble FasL (sFasL) in the serum of bladder cancer patients has been investigated. The data suggest that monitoring the level of sFasL and its cytotoxic activity may be the prognostic indicators for bladder cancer patients [28]. For OSMR gene, the hub gene of Luminal-papillary and Luminal-infiltrated may affect tumor classification, recurrence and overall survival rate [29]. A recently study has shown that its polymorphism is significantly associated to bladder cancer from West China Hospital of Sichuan University.

And for differential network on Neuroactive ligand-receptor interaction, related study has shown that Neuroactive ligand-receptor interaction may be involved in the development of lung cancer as a major pathway. It has been reported that the imbalance of the Neuroactive ligand-receptor interaction in lung cancer, and the Neuroactive ligand-receptor interaction is also related to nicotine dependence, and increases the risk of lung cancer [30]. Smoking is also an important risk factor for bladder cancer. Although it has been shown that the Neuroactive ligand-receptor interaction plays an important role in various stages of bladder cancer [31], the mechanism of its action on bladder cancer has not been revealed, and it is only widely recognized in neuro related diseases. In this work, according to the KEGG enrichment results, it may play a critical role in occurrence and molecular specificity of bladder cancer. Attention should be paid in the hub genes of differential networks based on Neuroactive ligand-receptor interaction.

## Discussion

Firstly, Gene regulatory networks have individual specificity. In order to further develop personalized treatment, building a differences network to determined subtype from normal to tumor may be to explain the change of individual regulatory relationship. However, it is difficult to get the data from normal state to disease state of a person, because it often does not have a patient’s normal state in advance. Besides, the sample size of personal data is small.

Secondly, Biological pathway information is incomplete. Biological pathways show the functional similarity of a group genes. From the perspective of regulatory relationships, it is naturally more meaningful to consider the group relationships than a single gene. However, biological pathways need further explanation and complement according to the published references. So more comprehensive, systematic, and specific biological processes in the human is also needed.

Finally, traditional bulk RNA-Seq reveals the average gene expression of an ensemble of cells, which cannot analyse the detailed states of individual cells. The development of single-cell RNA-Seq (scRNA-Seq) makes it possible to quantify the expression of single cells and analyze the detailed differences between single-cells. At present, there are some methods constructing gene regulatory network from single-cell data, such as SCODE [32], SCENIC [33], PIDC [34] and so on. Because scRNA-Seq can distinguish the detailed states of individual single-cell, it can accurately calculate the correlations of expression between genes. So it is an important research direction to construct gene regulatory network from single-cell data.

## Conclusions

In this paper, to reveal the differences between molecular subtypes of MIBC, we propose a complete analysis process, including differential expression analysis, pathway enrichment analysis and differential network construction. Through these analyses, some key genes were discovered. Furthermore, we observe that Cytokine-cytokine receptor interaction and Neuroactive ligand-receptor interaction almost appear in significantly different pathways between any two subtypes. Therefore, the relationship between these two pathways and bladder cancer should be explored. In the future, we will explore the subtype specificity with single-cell expression data.

## Methods

### Data

In this paper, the data are based on the transcriptome mRNA-seq files of The Cancer Genome Atlas (TCGA) program molecular typing results [13]. In [13], a comprehensive analysis of 412 cases of MIBC was reported, and five subtypes (Luminal-papillary: 142, Luminal-infiltrated: 78, Luminal: 26, Basal-Squamous: 142 and Neuronal: 20) were identified. We download subtypes data from Genomic data Commons (GDC) data portal, and preprocess the data into gene expression matrix. The workflow of data preprocessing is given in Fig. 2, and the specific steps are as follows:

**Step 1:** Extract the number of TCGA samples. The file name and corresponding TCGA sample number are stored in the metadata files from GDC. The sample number are extracted for subsequent replacement.

**Step 2:** Integrate data of the same subtype into an expression matrix and replace file names.There are multiple compressed files for each subtype of data, so we integrate the data of each subtype into a matrix and replace the file name with the sample number.

**Step 3:** Delete non-gene count data in the expression matrix. The first 5 rows of data in the integrated matrix are not count information of gene expression and should be deleted.

**Step 4:** Remove ENSEMBL version number of gene name. Delete the ENSEMBL version number of gene name, because it is not required in gene annotation.

**Step 5:** Screening paracancerous and poor quality samples. A patient may have multiple RNA-seq files where contain paracancerous samples and poor quality samples. Delete these samples because they will affect the accuracy of results.

**Step 6:** Filter non-expressed and low-expressed genes. Filtering non-expressed and low-expressed genes can improve the efficiency of subsequent differential expression analysis.

**Step 7:** Gene annotation and the coding gene extraction. Convert the gene name into gene symbol named by the HUGO Gene Nomenclature Committee(HGNC). Download the gene-annotated GTF file from ENSEMBL database for gene name conversion and extraction of encoded genes.

Finally, the expression matrix of each subtype and the combined expression matrix of five subtypes for differential expression analysis were obtained.

### Differential expression analysis

The goal of differential expression analysis is to find differentially expressed genes (DEGs) from two sample groups by analyzing gene expression data. The principle is to judge the existence of treatment effect by comparing intra-group difference and inter-group difference, and to prove that there are differentially expressed genes between groups. The intra-group difference is the error effect, and the inter-group difference is the sum of treatment effect and error effect. The essence of the test is that the treatment effect is equal to the difference between the average difference of inter-group and the average difference of intra-group which is greater than zero. Significance depends on the results of statistical tests, such as p_value or p_adjust. The smaller the value, the more significant the difference in gene expression among groups. In this case, samples of the same subtype belong to a group, and the aim is to find genes that are differentially expressed in different subtypes.

In this study, R package DESeq2 [35] is used for differential expression analysis. The starting point of DESeq2 analysis is the count matrix *K*, the rows correspond to genes and columns correspond to samples. The element *K*_*ij*_ represents the quantitative value of gene *i* expression in the sample *j*, and it is subject to negative binomial distribution. DESeq2 differential expression analysis uses the following generalized linear model: *K*_*ij*_∼*NB*(*μ*_*ij*_,*α*_*i*_), where *μ*_*ij*_ represents the mean, *α*_*i*_ represents the discrete factor which describes the degree of deviation of variance from the mean and defines the relationship between variance and mean, and the variance $v=\mu _{{ij}}+\alpha _{i}\mu _{{ij}}^{2}$.

In addition to count matrix, the input data DESeq2 also includes group matrix and difference comparison matrix. Group matrix stores the group information. Difference comparison matrix corresponds group information and their sample. The whole process of DESeq2 differential expression analysis involves three steps: constructing dds object for storing data, invoking DESeq function for differential expression analysis, and using result function to extract difference analysis results, which returns a result table of data such as *log*2*FoldChange* (differential multiple of *log*_2_), p_value, p_adjust and so on. *log*2*FoldChange* indicates the difference multiple of the average expression amount corresponding to the two groups, and the p_adjust indicates the result of the multiple check correction p_value.

### KEGG pathway enrichment analysis

The goal of the KEGG enrichment analysis is to annotate the list of differentially expressed genes into the pathways of the KEGG PATHWAY database and screen out significant pathways based on statistical tests. In this context, the enrichment analysis used the R package clusterProfiler [36] to obtain the pathway annotation information by invoking KEGG API. The principle is to intersect the DEGs with the gene sets of a pathway in KEGG PATHWAY database, find common genes and count them, and finally use statistical tests to evaluate whether the observed counts are significantly. The statistical test method used by clusterProfiler is Fisher’s exact test, which calculates p_value based on hypergeometric distribution:

1 $$\begin{array}{@{}rcl@{}} p\_value={\sum\nolimits}_{i=m}^{M}\frac{C_{M}^{i}C_{N-M}^{n-i}}{C_{N}^{n}} \end{array} $$

where *N* is the total number of human genes in KEGG PATHWAY database, *M* is the number of genes belonging to a pathway in KEGG PATHWAY database, *n* is a list of differentially expressed genes, *i* is the number of *M* in *n*, and p_value indicates the probability of randomly extracting *n* genes from *N* genes. The clusterProfiler uses the BH [37] method to adjust the p_value to get p_adjust.

### Differential network

The WD-trace model assumes that the gene expression data of *p* genes in two groups are subject to two multivariate Gaussian distributions, the formulas can be expressed as:

2 $$\begin{array}{@{}rcl@{}} X = (X_{1},...,X_{p})^{T} \sim N(0,\Sigma_{X}) \end{array} $$

3 $$\begin{array}{@{}rcl@{}} Y = (Y_{1},...,Y_{p})^{T} \sim N(0,\Sigma_{Y}) \end{array} $$

Define the precision matrix *Θ*=*Σ*^−1^, and the differential network is defined by $\Delta =\Theta _{Y}-\Theta _{X}=\Sigma _{Y}^{-1}-\Sigma _{X}^{-1}$. Suppose that *A*=(*A*_*ij*_)∈*R*^*p*×*p*^ is a *p*×*p* matrix, $ A=\sum _{i,j=1}^{p}|A_{{ij}} |_{1}$ will denote the elementwise L1 norm. Let <*A,B*>=*tr*(*AB*)^*T*^ be the inner production. The D-trace loss function is defined as follows:

4 $$ \begin{aligned} L_{D}(\Delta;\hat{\Sigma}_{X},\hat{\Sigma}_{Y})&=\frac{1}{4}(<\hat{\Sigma}_{X}\Delta,\Delta\hat{\Sigma}_{Y}>+\\&<\hat{\Sigma}_{Y}\Delta,\Delta\hat{\Sigma}_{X}>) -<\Delta,\hat{\Sigma}_{X}-\hat{\Sigma}_{Y}>\end{aligned}  $$

where the sample covariance matrix $\hat {\Sigma }_{X}=\frac {1}{n_{X}}X^{T}X$ is the estimator of covariance matrix *Σ*_*X*_, and the sample covariance matrix $\hat {\Sigma }_{Y}=\frac {1}{n_{Y}}Y^{T}Y$ is the estimator of covariance matrix *Σ*_*Y*_. This loss function is designed for the solving goal $\hat {\Delta }$. If $\Delta =\Sigma _{Y}^{-1}-\Sigma _{X}^{-1}, \Sigma _{X}\Delta \Sigma _{Y}-(\Sigma _{X}-\Sigma _{Y})=0 $ and *Σ*_*Y*_*ΔΣ*_*X*_−(*Σ*_*X*_−*Σ*_*Y*_)=0 are satisfied, then $\frac {1}{2}(\Sigma _{X}\Delta \Sigma _{Y}+\Sigma _{Y}\Delta \Sigma _{X})-(\Sigma _{X}-\Sigma _{Y})=0 $ can be obtained. The gradient parameter of D-trace loss can be defined as:

5 $$ \nabla{L_{D}}= \frac{1}{2}(\Sigma_{X} \Delta \Sigma_{Y}+\Sigma_{Y}\Delta\Sigma_{X})-(\Sigma_{X}-\Sigma_{Y})  $$

where the minimum of *L*_*D*_ is taken at $\hat {\Delta }=\hat {\Sigma }_{Y}^{-1}-\hat {\Sigma }_{X}^{-1}$. The static regulatory network is constructed by TRRUST public dataset and defined as *S*=(*S*_*ij*_)∈{0,1}^*p*×*p*^,where *S*_*ij*_=1 indicates that there is a relationship between gene *i* and *j*. Meanwhile, the weight matrix *W* is defined to make the penalty weight be *w*∈(0,1) if *S*_*ij*_=1, and the weight is 1 if *S*_*ij*_=0,*W*_*ij*_ is defined as.

6 $$ W_{{ij}}=\left\{ \begin{aligned} w, S_{{ij}}=1 \\ 1, S_{{ij}}=0 \end{aligned} \right.  $$

In this way, the static network and gene expression data can be combined. In fact, there are only a few edges in differential network, because *Δ* is sparse. In this view, WD-trace could add the weighted lasso penalty to the D-trace loss function, so a new model as follow:

7 $$ \Delta= \mathop{\arg\min_{\Delta}}\{ L_{D}(\Delta;\hat{\Sigma}_{X},\hat{\Sigma}_{Y}) +\lambda\sum_{1\leq i,j\leq p}W_{{ij}}|\Delta_{{ij}}| \}  $$

where *λ* is a nonnegative tuning parameter. In reality, two genes are more likely to connect in differential network if they are linked in static regulatory network. For the penalty weight *w* of two genes, the smaller the *w*, the more likely they are connected in static estimated GRN. Since there are a few edges in TRRUST database for gene in Neuroactive ligand-receptor interaction and Cytokine-cytokine receptor interaction, we set *w* equal to 0.1. In addition, *λ* is selected using stability selection method, StARS [38]. The threshold parameter of StARS is set as *β*=0.005 and the number of sample subsets is set as *S*=20. Finally, the model (7) is solved by accelerated proximal gradient descent method[10, 39]. The proximal gradient method can be written as follows:


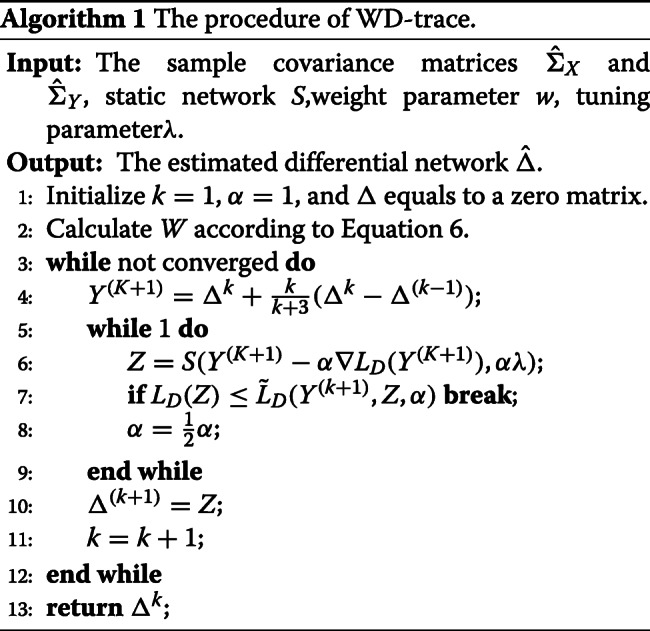

8 $$ \begin{aligned} \mathbf{prox}_{p}(A)&=\mathop{\arg\min_{\Delta}}\lambda\sum_{1\leq i,j\leq p}W_{{ij}}|\Delta_{{ij}}|+\frac{1}{2}||\Delta-A||_{2}^{2}\\ &=sign(A)max(|A|-\lambda,0)\\ &=S(A,\lambda) \end{aligned}  $$

The function $\tilde {L}_{D}$ is given by

9 $$ \begin{aligned} \tilde{L}_{D}(Y,Z,\alpha)&=L_{D}(Y)+tr(\nabla{L_{D}(Y)}(Z-Y))\\ &+\frac{1}{2\alpha}||Z-Y||_{2}^{2} \end{aligned}  $$

The procedure of WD-trace is shown in Algorithm 1.

In this study, WD-trace method is used to construct the differential network to infer the regulatory relationships between genes and obtain the hub gene. In this study, the hub genes are the top 10 genes with the highest degree in GRNs.

## Data Availability

The dataset about this manuscript can be downloaded from The Cancer Genome Atlas (TCGA) program. The accession number for the RNA-seq data for human bladder cancer cell lines reported in this paper is GEO: GSE97768.

## Abbreviations

GRN: Gene regulatory network
MIBC: Muscle-invasive bladder cancer
GGMs: Gaussian Graphical Models
TRRUST: Transcriptional Regulatory Relationships Unraveled by Sentence-based Text-mining
GO: Gene Ontology
KEGG: Kyoto Encyclopedia of Genes and Genomes
TCGA: The Cancer Genome Atlas
GDC: Genomic data commons
DEGs: Differential expressed genes
